# Author Correction: Similar levels of emotional contagion in male and female rats

**DOI:** 10.1038/s41598-020-67128-w

**Published:** 2020-07-23

**Authors:** Yingying Han, Bo Sichterman, Maria Carrillo, Valeria Gazzola, Christian Keysers

**Affiliations:** 1 Netherlands Institute for Neuroscience, Royal Netherlands Academy of Arts and Sciences, Amsterdam, the Netherlands; 2 Department of Psychology, Faculty of Social and Behavioural Sciences, University of Amsterdam (UvA), Amsterdam, The Netherlands

Correction to: *Scientific Reports* 10.1038/s41598-020-59680-2, published online 17 February 2020

The original version of this Article contained an error in the name of the author Maria Carrillo, which was incorrectly given as Carrillo Maria. This has now been corrected in the PDF and HTML versions of the Article.

